# Mechanical analysis of the structure of longwall mining hydraulic support

**DOI:** 10.1177/0036850420936479

**Published:** 2020-08-10

**Authors:** Tengda Li, Jiren Wang, Kun Zhang, Chunhua Zhang

**Affiliations:** 1 School of Safety Science and Engineering, Liaoning Technical University, Fuxin, China; 2 College of Mechanical and Electronic Engineering, Shandong University of Science and Technology, Qingdao, China

**Keywords:** Hydraulic support, group support, longwall mining operations, offset load, static analysis

## Abstract

Existing studies of the structural strength of longwall mining hydraulic support are mainly focused on the force acting on individual supports instead of the general mechanical characteristics of the support group in a fully mechanized coal seam working face. This study combines theoretical analyses and experiments to investigate the mechanical characteristics of a longwall mining hydraulic support group and the stiffness of key support components under different working conditions. The theory of a beam on an elastic foundation was applied to construct a mechanical model for the hydraulic support group. The location and the size of loads on the top beam were determined. Field tests yielded data on the deflection of the roof and loading on the support group along the working face, where the stiffness of end supports varies. The transverse load distribution of the top beam and the offset loading coefficient at different locations along the working-face direction were obtained. A three-dimensional model was constructed for the support group while assembling virtual hydraulic supports using modern virtual modeling theories and methods. Finite element analysis was used to analyze the strength of the hydraulic support. The weakest areas of key components were found to be pinholes connecting the column cylinder to the base and roof of the mine. These results can be applied to achieve secure and stable operations of hydraulic supports in the working face of a thin coal seam, thereby improving the safety and production efficiency of mining operations.

## Introduction

Understanding the mechanical characteristics and stiffness of hydraulic supports is vital to ensuring stable operation in a mining well. This is especially true for working faces of thin coal seams, where equipment replacement and maintenance are difficult. Analysis of support components should be performed according to mechanical design theories and methods using modern engineering design and analysis software. The results are of great significance to coal seam mine design, the prediction of the stiffness of weak parts, and optimization of the support structure.^1,2^

Zhou et al.^3^ used the ZY12000/28/64 roof cover hydraulic supports as primary reference objects and constructed a kinetic equation by applying the Lagrange equations of the second kind. The kinetic equation was then used to analyze the quality, initial height, and fracture point of the main roof. The results of this study show that these parameters have a profound influence on the dynamics of hydraulic support. Liang et al.^4^ constructed a model using ADAMS simulation software. A column hydraulic cylinder, balance hydraulic cylinder, rear connecting rod, and shield beam were treated as simplexes in the model to simulate the bearing capacity of the structure. Xu et al.^5,6^ investigated the bearing capacity of a two-column shield hydraulic support using a plane bar support system model. After analyzing the support–roof connection and the mechanically balanced zone of the support, the author defined and classified the bearing capacities and highlighted their influences. The coupling relationship between the hydraulic support and the surrounding rocks in a mine was also analyzed from a stiffness coupling perspective. Xu et al.^7^ further conducted resistance-increasing stiffness tests to determine the stiffness coupling relationships between the hydraulic support and the coal wall, roof, and floor, respectively. Coupled equations were developed to improve the calculation of the supporting strength required for equipment and to investigate stiffness coupling. Yang et al.^8^ developed an overlying strata breakage prediction experiment platform, which was used to simulate the dynamic load impact in a shallow working face with thin bedrock. A breakage model of the roof strata was proposed based on the elastic foundation beam model to calculate the relative distance between the main roof’s breakage and the coal wall and analyze the sphere of dynamic loading. In 2017, Wan et al.^9^ proposed that loading of the impact-affected hydraulic support shield beam would then influence the support movement trend, the states at which forces are applied, and the structure of the shield beam.

In the above-mentioned studies, the main consideration was the effect of vertical loads (along the direction of the working face) on the hydraulic support. However, relatively few reports seek to understand how lateral loads alter hydraulic support structures. This study investigates the impacts of lateral loads by analyzing the structural strength of the hydraulic support based on the mechanical characteristics of the support group. ANSYS simulation software was employed to analyze the stresses and the deformations of key components, such as the top beam, base, and column, under offset-loading conditions. The weak portions of these components were identified under different working conditions, and field tests were conducted to verify the feasibility of the research methods described in this article.

## Working condition analysis of hydraulic support group

**Figure 1.**
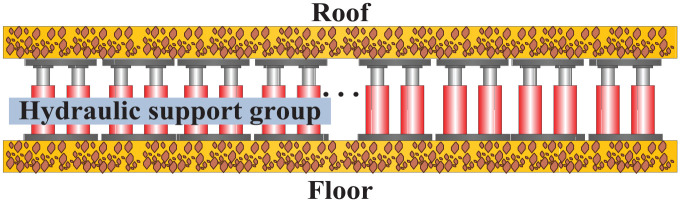
Schematic diagram of the support group composed of hydraulic supports.

The working face of a mine generally employs a two-column hydraulic structure to support the roof. A standing shield-type hydraulic support controls the roof at the front and rear ends. The hydraulic supports can move automatically based on inputs from an electro-hydraulic control system. When the roof breaks as the working face passes through a fractured zone or when the rib spalling is so wide that the roof quality easily deteriorates, the hydraulic supports are moved using manual and automatic methods. Tens to hundreds of hydraulic supports combine to form the supporting system of the working face. As shown in Figure 1, these supports form a support group to support the roof rock along the working face in a fully mechanized mining seam.^10^

**Figure 2.**
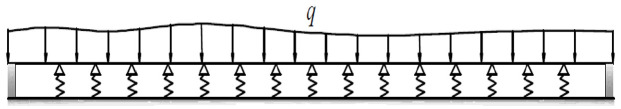
Rock beam model of an elastic support group.

A working hydraulic support is usually characterized by increasing resistance and pressure, constant-resistance bearing, and overflow at constant pressure. A hydraulic support can be thought of as an elastic body with stiffness K . Xu and Wang^10^ considered the lateral side of the roof as the beam body and the hydraulic support group under the roof as multiple elastic bodies with stiffness K . The hydraulic support system at the working face can then be modeled as a beam body lying on an elastic foundation, as shown in Figure 2.

The roof of the working face is also supported by the roadways in a fully mechanized coal seam. However, the roadway deforms as it bears loads from the roof. Therefore, the roadway can be included as the base in the simplified model shown above. When the roadway bearing load is deformed in the vertical direction, it can also be regarded as an elastic foundation with stiffness, K . Because of the horizontal bending of the roof beam in the roadway, the two ends of the beam can then be considered as fixed elastic ends with stiffness, $K_{\theta }$ . As a torque, *M_e_* acts on the beam body in the roadway, an angle θ results, which can be calculated using the equation $\theta =M_{e}/K_{\theta }$ . The hydraulic support, which is considered to be an elastic body, is between the roof and the floor. In this study, we assume that the floor is too rigid to be deformed, the load on the hydraulic support is mainly from the roof, and the roof load is uniformly distributed.

## Stress analysis of the hydraulic support group

### Stress analysis based on elastic beam

The working-face roof rock in a fully mechanized coal seam is supported by hundreds of hydraulic structures packed closely together. As the immediate gap between adjacent hydraulic supports of the beam is negligible, the support group can be considered an elastic body with stiffness, K . We can consider the lateral side of the roof as the beam body, and the support group under the roof as multiple elastic bodies with stiffness.^10^ We set the midpoint of the roof as the origin of our coordinate system.

**Figure 3.**
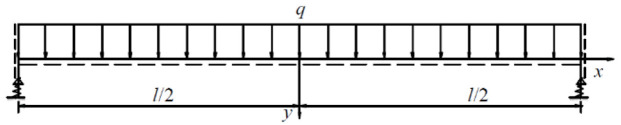
Elastic foundation beam model.

According to the research in the literature,^11–13^ it is proved that the mechanical properties of the left and right roadway of the fully mechanized mining face are basically the same, and the loads of the hydraulic support and the roof are basically symmetrically distributed relative to the center line of the working face. We assume that the mechanical characteristics of the left and right roadways are the same and the loads on the hydraulic support and the roof load are symmetrically distributed against the central line of the working face.^10^ The supporting system in the working face is simplified to an elastic foundation beam, as illustrated in Figure 3.

The stiffness of the support can be expressed as

(1) K=kBL

where B represents the center distance of the support, L is the length of the upper beam, and k is the coefficient of subgrade reaction that indicates the allowable settlement of the beam.

If we define the uniformly distributed load on the roof as q and the load distribution of the hydraulic support against the roof as −kv , the loads imposed on the roof can then be calculated as q−kv .^14,15^ Based on the assumption of elastic foundation, the following equilibrium equation can be obtained for each unit width of the roof

(2) $$\mathrm{EI}v^{\left(4\right)}+\mathrm{kv}=q$$

where v is the settlement amount of the roof for any unit support in the rock beam, EI is the bending stiffness, and q is the uniformly distributed load on the roof.

The general solution for equation (2) is given as follows

(3) $$v(x)=v_{0}V_{0}(\alpha x)+\frac{\theta_{0}V_{1}(\alpha x)}{\sqrt{2}\alpha }+\frac{M_{0}V_{2}(\alpha x)}{2\alpha^{2}\mathrm{EI}}+\frac{N_{0}V_{3}(\alpha x)}{2\sqrt{2}\alpha^{3}\mathrm{EI}}+\frac{q}{k}\left[1-V_{0}(\alpha x)\right]$$

where $v_{0}$ is the deflection and $M_{0}$ is the bending moment at the rock beam midpoint.

Due to structural symmetry, both turning angle and shear force at the rock beam midpoint are 0, that is, $\theta_{0}=N_{0}=0$ , which is substituted into equation (3) to obtain the torsion curve of the rock beam (roof) as^16^

(4) $$v(x)=C_{0}V_{0}(\alpha x)+C_{2}V_{2}(\alpha x)+\frac{q}{k}$$

where $C_{0}=v_{0}-\frac{q}{k}$ and $C_{2}=\frac{M_{0}}{2\alpha^{2}\mathrm{EI}}$ , $\alpha =\sqrt[{4}]{\frac{k}{4\mathrm{EI}}}$ .

$V_{i}\left(\mathrm{ax}\right)\left(i=0\sim 3\right)$ is the Puzyrevsky (Rus. Пузыревский) function, that is, when *i* = 0–3:

(5) $$\begin{array}{c} V_{0}(\alpha x)=\mathrm{ch}(\alpha x)\cos(\alpha x)\\ V_{1}(\alpha x)=\frac{1}{\sqrt{2}}\left[\mathrm{ch}(\alpha x)\sin(\alpha x)+\mathrm{sh}(\alpha x)\cos(\alpha x)\right]\\ V_{2}(\alpha x)=\mathrm{sh}(\alpha x)\sin(\alpha x)\\ V_{3}(\alpha x)=\frac{1}{\sqrt{2}}\left[\mathrm{ch}(\alpha x)\sin(\alpha x)-\mathrm{sh}(\alpha x)\cos(\alpha x)\right]\\ {V\prime }_{0}\left(\alpha x\right)=-\sqrt{2}\alpha V_{3}\left(\alpha x\right);\\ {V\prime }_{1}\left(\alpha x\right)=\sqrt{2}\alpha V_{0}\left(\alpha x\right);\\ {V\prime }_{2}\left(\alpha x\right)=\sqrt{2}\alpha V_{1}\left(\alpha x\right);\\ {V\prime }_{3}\left(\alpha x\right)=\sqrt{2}\alpha V_{2}\left(\alpha x\right);\\ V_{0}(0)=1;\;V_{1}(0)=1;\;V_{2}(0)=1;\;V_{3}(0)=1; \end{array}$$

In terms of the load imposed on the hydraulic support group, we assumed that the roofs on the left and right sides of the roadway are stiffened to the elastic foundation. The boundary conditions can then be obtained from the following equation: $K_{\theta 1}=K_{\theta 2}\to \infty$ . If u=al/2 , and the end turning angle is v′(−l/2)=v′(l/2)=0 , $K_{1}v\left(-l/2\right)=-\mathrm{EIv}\prime \prime \prime \left(-l/2\right)$ then the following equation can then be derived from equation (3)^10^

(6) $$\left\{ \begin{array}{c} K\left[C_{0}V_{0}\left(u\right)+C_{2}V_{2}\left(u\right)+\frac{q}{k}\right]=-2\sqrt{2}\mathrm{EI}\alpha^{3}\left[C_{0}V_{1}\left(u\right)+C_{2}V_{3}\left(u\right)\right]\\ C_{0}V_{3}\left(u\right)-C_{2}V_{1}\left(u\right)=0 \end{array}\right.$$

From the second formula in equation (6), we can obtain the following relation: $C_{2}=\frac{C_{0}V_{3}\left(u\right)}{V_{1}\left(u\right)}$ , which is then substituted into the first formula of equation (6) to get

(7) $$\mathrm{Ku}\left[C_{0}V_{0}\left(u\right)V_{1}\left(u\right)+C_{2}V_{2}\left(u\right)V_{3}\left(u\right)+\frac{qV_{1}\left(u\right)}{k}\right]=-\frac{\sqrt{2}}{4}\left[C_{0}{V_{1}}^{2}\left(u\right)+C_{2}{V_{3}}^{2}\left(u\right)\right]$$

Substituting equation (7) into the Puzhevsky function leads to

(8) $$C_{0}=-\frac{4\sqrt{2}\mathrm{Kqu}V_{1}(u)}{k\left[2\mathrm{Ku}(\mathrm{sh}2u+\sin2u)+\mathrm{kl}(\mathrm{ch}2u-\cos2u)\right]}$$

Let $A=\frac{\mathrm{kl}}{2\mathrm{Ku}}f\left(u\right)$ , and $f\left(u\right)=\frac{\mathrm{ch}\left(2u\right)-\cos\left(2u\right)}{\mathrm{sh}\left(2u\right)+\sin\left(2u\right)}$ , then, substitution into the Puzhevsky function provides *C*_0_ as

(9) $$C_{0}=-\frac{q}{k}\frac{\varphi (u)}{1+A}$$

where $\phi \left(u\right)=\frac{2\left(\mathrm{ch}u\sin u+\mathrm{sh}u\cos u\right)}{\mathrm{sh}\left(2u\right)+\sin\left(2u\right)}$ .

Again, substituting this equation to obtain $C_{2}$ as

(10) $$C_{2}=-\frac{q}{k}\frac{\psi (u)}{1+A}$$

where $\psi \left(u\right)=\frac{2\left(\mathrm{ch}u\sin u-\mathrm{sh}u\cos u\right)}{\mathrm{sh}\left(2u\right)+\sin\left(2u\right)}$ .

Finally, substituting $C_{0}$ and $C_{2}$ into equation (6) yields the following relation

(11) $$v(x)=\frac{q}{k}\left[1-\frac{\varphi (u)V_{0}(\alpha x)+\psi (u)V_{2}(\alpha x)}{1+A}\right]$$

### Roof load

**Figure 4.**
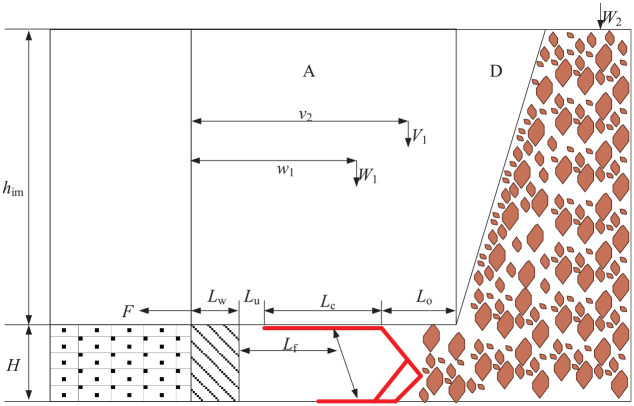
Roof load model of beam.

The roof load model of the beam is shown in Figure 4.

The weight, $W_{1}$ , of the roof acting directly on the support is

(12) $$\begin{array}{c} W_{1}=W_{A}+W_{D}=h_{\mathrm{im}}\gamma_{\mathrm{im}}\mathrm{BL}\cos\beta +\frac{h_{\mathrm{im}}^{2}}{2}\gamma_{\mathrm{im}}B\tan\phi \cos\beta \\ =h_{\mathrm{im}}\gamma_{\mathrm{im}}B\left(L+\frac{h_{\mathrm{im}}}{2}\tan\phi \right)\cos\beta \end{array}$$

where B is the distance between the centerlines of two adjacent hidden supports, ϕ is the roof caving angle, $h_{\mathrm{im}}$ is the caving height, and β is the inclination angle of the coal seam.^17,18^

The length of beam, L , after coal shearing can be obtained as follows

(13) $$L=L_{w}+L_{u}+L_{c}+L_{o}$$

where *L_u_* is the tip to face distance, *L_w_* is the cut-off depth, *L_c_* is the length of the top beam of the support, and *L_o_* is hanging roof length.

The length of the beam, L , before coal shearing is

(14) $$L=L_{u}+L_{c}+L_{o}$$

The location of $W_{1}$ acting on the beam can then be determined using the following equation

(15) $$W_{1}=\frac{L+h_{\mathrm{im}}\tan\phi }{2}$$

The bearing locations on the beam are determined by the stiffness of the roof rocks over the top beam. When the coefficient of the subgrade reaction for hard rock is sufficiently large to meet the condition $W_{1}>L_{f}$ , the load at the beam rear is significantly higher than that at the front end. In contrast, when the coefficient of the subgrade reaction for hard roof rocks is small enough to meet the condition $W_{1}<L_{f}$ , the load at the front end of the beam is considerably higher than that at the rear end.

## Hydraulic support group load calculation

**Figure 5.**
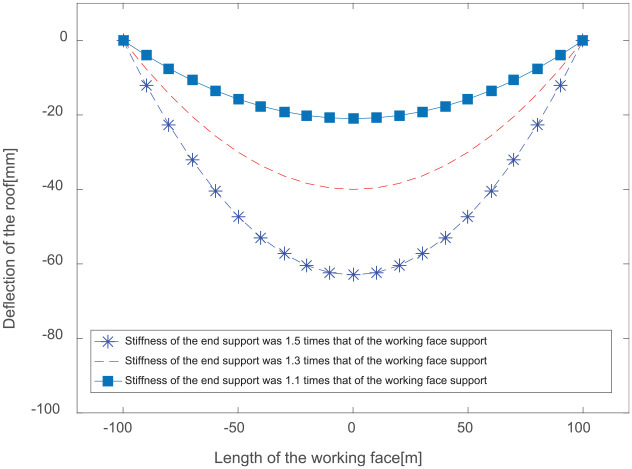
Deflection of the roof with varying end support stiffness values.

**Figure 6.**
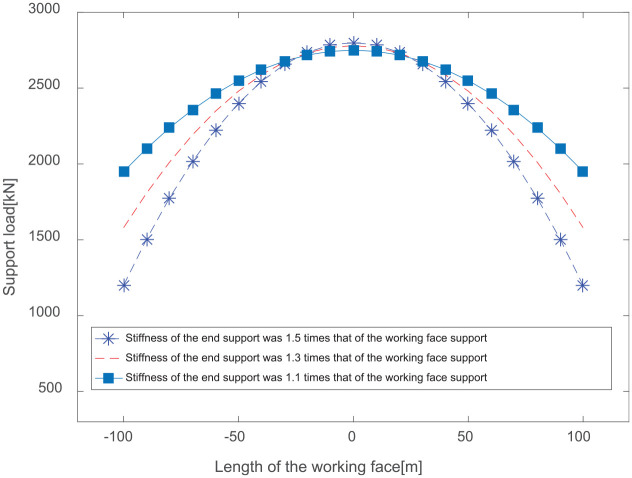
Supporting force of a hydraulic support group with varying end support stiffness values.

Roof deflections and loads on the hydraulic support group along the working face can be obtained using our mechanical model under different-end support stiffness conditions. The stiffnesses of the front-end support and the rear-end support were set to 1.1, 1.3, and 1.5 times the working stiffness of the hydraulic support, respectively. The roof thickness is 8.5 m, the mining height is 4.5 m, and the distance between the support centerlines is 1.75 m. The rock bulk density is 25 kN/m^3^, the elastic modulus of the roof is 40 GPa, the support stiffness is 200 MN/m, and the length of the longwall fully mechanized mining face is 250 m. The roof caving angle is 30°, and the caving height is 36 m. The inclination angle of the coal seam is 0°. The distance between the coal mining end faces is 0.5 m, while the cut-off depth is 0.8 m. The length of the roof support beam is 5.2 m, and the length of hanging roof is 1.5 m. By obtaining the stiffness conditions of the face-end support, the conditions of the roof deflection are as shown in Figure 5; the supporting force of the longwall mining hydraulic support group is shown in Figure 6.

**Figure 7.**
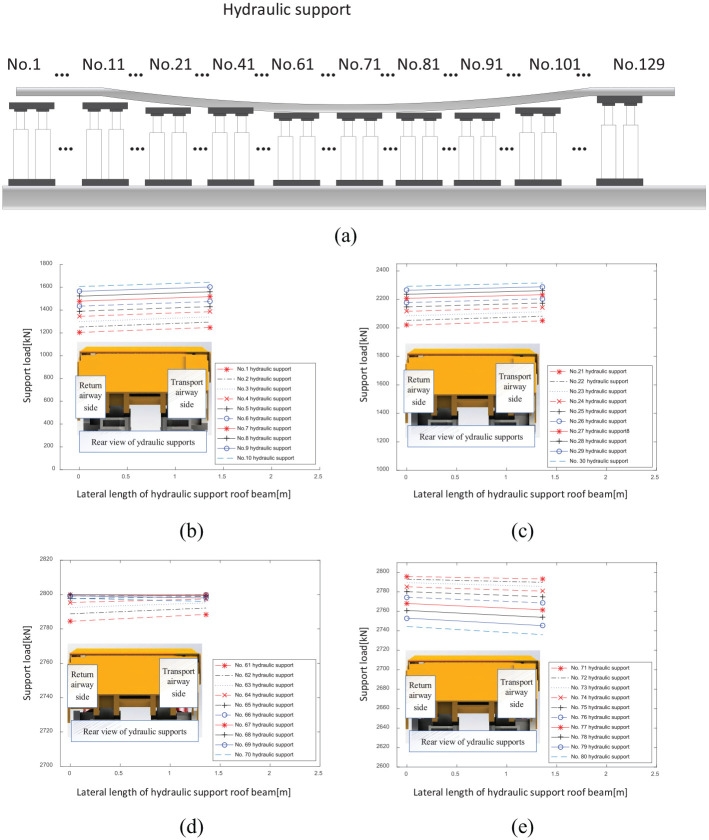
Top beam load of hydraulic support at different working face positions. (a) Serial number definition of hydraulic support on working faces, (b) hydraulic supports 1–10, (c) 21–30, (d) 61–70 and (e) 71–80.

The above analyses yield the roof deflections and supporting force of the hydraulic support group under different-end support stiffness conditions. The results indicate that loads and offset loads on the top beam of the hydraulic support vary at different locations on the working face. The closer the location is to the middle of the working face, the larger the load on the beam, and the smaller the offset load. The results are shown in Figure 7.

**Figure 8.**
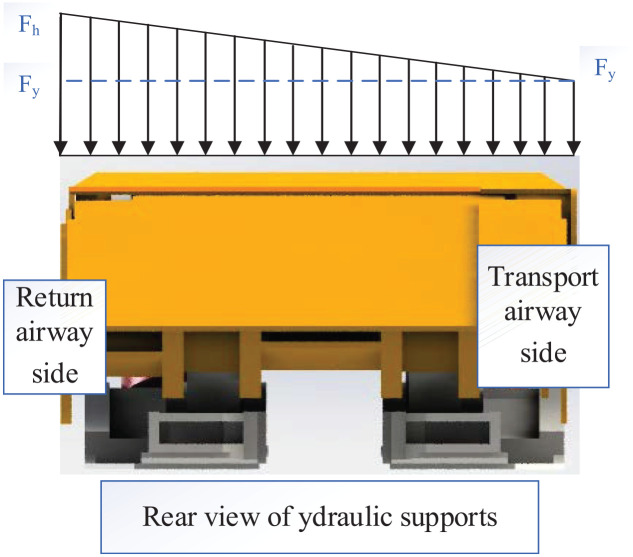
Schematic diagram of loading eccentric coefficient of the load on the top beam.

The coefficient, $w_{p}$ , is introduced to represent the offset loading on the top beam of the hydraulic support

(16) $$w_{p}=\frac{F_{h}-F_{y}}{F_{h}}$$

where *$F_{h}$* is the load on the return airway side of the top beam and $F_{y}$ is the load on the transport airway side of the top beam, as shown in Figure 8.^19^

**Figure 9.**
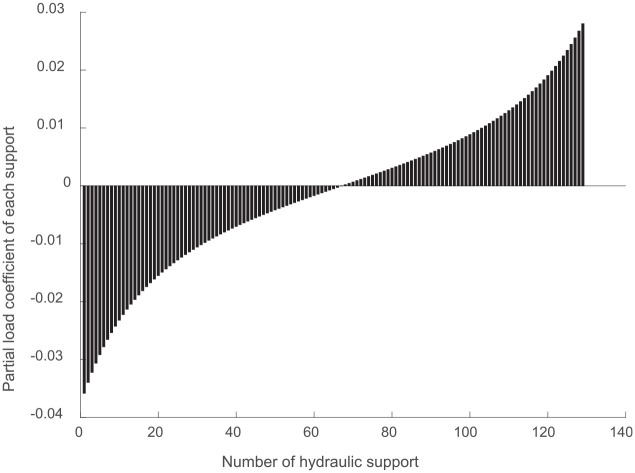
Eccentric coefficient of the load on the top beam.

The offset loading coefficients of the hydraulic supports along the working face were calculated, as shown in Figure 9.

## Mechanical analysis of key components of the hydraulic support

### Boundary conditions

The mechanical characteristics and structural strength of the hydraulic support bearing offset loads from the roof were investigated under working conditions where a support group was implemented. The loading results presented in the section “Stress analysis of the hydraulic support group” were used as the load and boundary conditions for finite element simulations.

**Figure 10.**
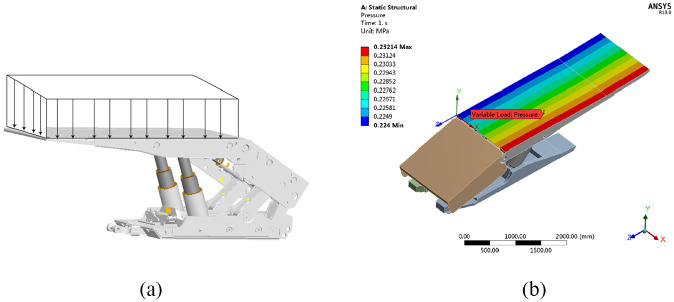
Finite element simulation of working condition settings. (a) Load type of working condition no. 1 and (b) load of working condition no. 1 imposed on the simulation model.

Hydraulic supports at three typical working locations of the return airway, namely, the 10th, 30th, and 65th

supports, were selected for the study. The results were then extended to all hydraulic supports of the working face. The loads calculated in the section “Stress analysis of the hydraulic support group” for these three hydraulic supports were applied to the top beam of the support model as uniformly distributed loads. Working condition nos. 1, 2, and 3 were defined for the three supports, respectively. The schematic diagram of the load application is shown in Figure 10.

Under working condition no. 1, $F_{y1}=1247.6\;\mathrm{kN}$ and $F_{h1}=1204.5\;\mathrm{kN}$ ; under working condition no. 2, $F_{y2}=2415.8\;\mathrm{kN}$ and $F_{h2}=2394.2\;\mathrm{kN}$ ; and under working condition no. 3, $F_{y3}=2797.3\;\mathrm{kN}$ , $F_{h3}=2801.5\;\mathrm{kN}$ , and $F_{m}=2801.5\;\mathrm{kN}$ . These values were applied to structural strength fine element analysis of the hydraulic support under the three working conditions.

### Results of finite element analysis

**Figure 11.**
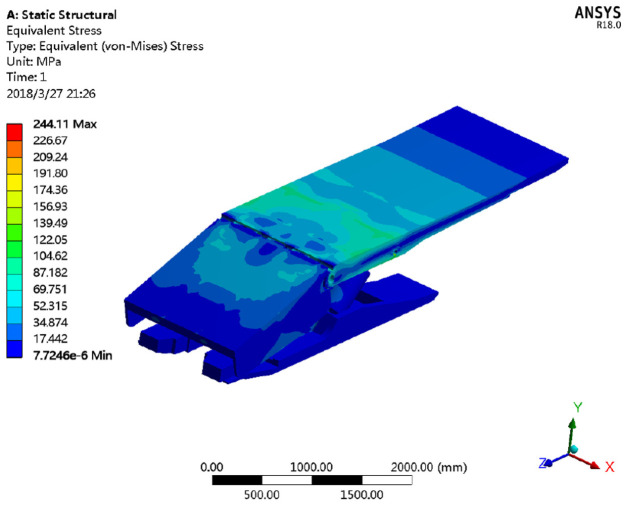
Whole frame stress cloud diagram of the hydraulic support.

Finite element simulation of hydraulic support strength was conducted after defining the model attributes, grid generation, and boundary conditions. Simulation results for working condition no. 1 are shown in detail as an example. The results of working condition nos. 2 and 3 and a comparison of simulation results and test results are presented in the section “Mechanical analysis of key components of the hydraulic support.” A cloud chart of the stresses on the hydraulic support under working condition no. 1 is shown in Figure 11.

#### Stresses and deformations of the top beam

**Figure 12.**
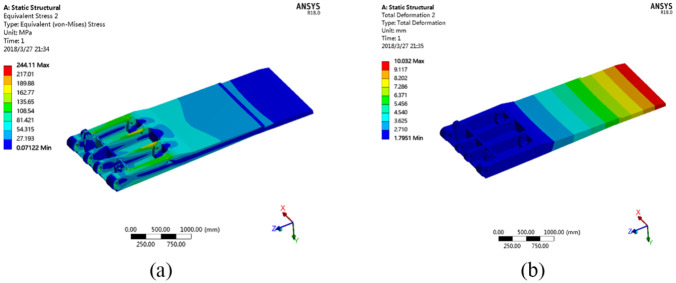
Distribution of stress and deformation of the hydraulic support top beam. (a) Stress distribution cloud chart and (b) deformation distribution cloud chart.

The stresses and deformations of the top beam of the hydraulic support are shown in Figure 12.

As shown in Figure 12(a), the stress of the top beam is mainly distributed around the column socket and middle rib plate, reaching a peak of 244.11 MPa near the column socket. The stress on the left column socket is higher than that on the right column socket due to the offset load. The stress at the upper left of the middle rib plate of the beam is 156.21 MPa, which is relatively high, while the stress on the right column socket is 135.88 MPa. The difference between the stress on the left and right column sockets is 20.33 MPa. In actual applications, a piston rod is usually on the top beam of the hydraulic support after the column pushes the pin shaft up until the latter is fractured. The theoretical analysis presented here is in good agreement with actual support structures. The column socket of the beam is the major load-bearing component when loads are applied to the roof. Hence, the strength of the column socket has a direct effect on the longevity of the hydraulic support. Stresses on the remaining portions of the top beam are not high, never exceeding the 690 MPa bearing yield limit of Q690, the raw material of the beam.

As shown in the deformation distribution cloud chart, the closer the top beam is toward its rear end, the smaller the deformation, and vice versa. The maximum deformation is 10.032 mm.

#### Stresses and deformations of the base

**Figure 13.**
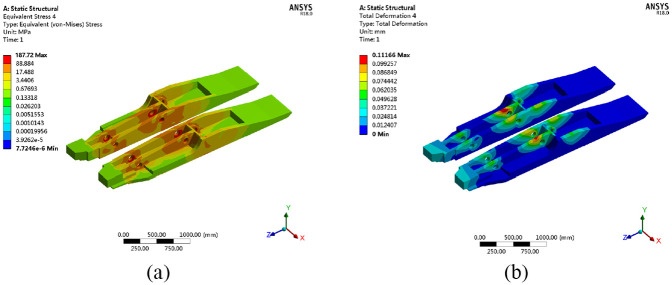
Distribution of stress and deformation of the hydraulic base. (a) Stress distribution cloud chart and (b) deformation distribution cloud chart.

Figure 13 shows the stresses and deformations of the base of the hydraulic support under working condition no. 1.

As shown in the stress distribution cloud chart, the pinholes connecting the cylinder and the base, as well as those connecting the rear connecting rod and the base, are where stresses are concentrated and significant deformations occur. The deformation and stress-concentrated areas on the left are larger than those on the right side of the base. The stress on the pinhole of the column reaches 187.72 MPa with a deformation of 0.11 mm. The deformation of the pinhole that connects the rear connecting rod and the base is 0.06431 mm. The major bearing component of the base is made from Q550, which has a yield stress of 550 MPa. This shows that the deformation of the major bearing component is within the elastic deformation range under this working condition, thus causing no permanent deformation of the base.

#### Stresses and deformations of the column cylinder

**Figure 14.**
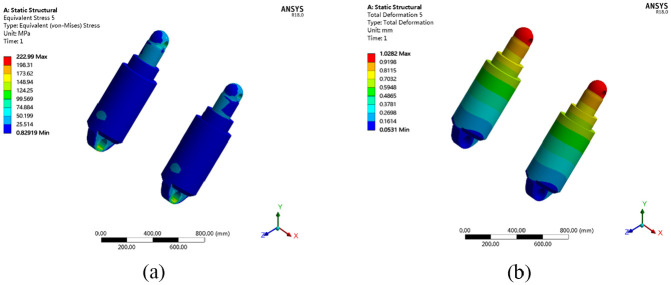
Distribution of stress and deformation of column cylinder. (a) Stress distribution cloud chart and (b) deformation distribution cloud chart.

The distribution of stresses and deformations of the column cylinder under working condition no. 1 are shown in Figure 14.

The maximum equivalent stress on the body of the column cylinder block is 222.99 MPa under working condition no. 1, with a deformation of 1.0282 mm. Under the influence of an offset load, the difference in the maximum equivalent stresses and deformation discrepancy between the left and right column cylinders are 19.48 MPa and 0.1446 mm, respectively. As shown in Figure 14, the stresses and deformations of the column cylinder block are within the limiting values of the raw material of the cylinder under working condition no. 1.

## Test result analyses

**Figure 15.**
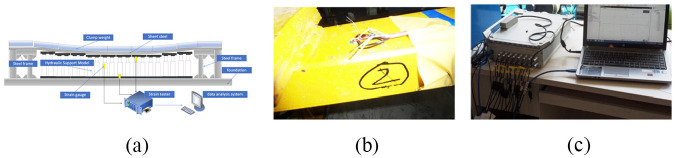
Hydraulic support bearing characteristics experiment. (a) Schematic of experimental model, (b) adhesive strain gauge at key parts, and (c) strain test data acquisition system.

Due to the requirements and limitations of underground explosion protection in coal mines, test instruments cannot be used in underground mining faces. Therefore, we establish a similar simulation test bench on the ground. Our test simulates the actual working conditions of the underground mining face, tests the bearing characteristics of the hydraulic support, and then converts the load characteristics of the support group to compare with the theoretical analysis. A schematic of the experimental model is shown in Figure 15(a). A strain gauge is attached to the corresponding position of the hydraulic support test model, as shown in Figure 15(b), and the strain test data acquisition system used is shown in Figure 15(c).

**Figure 16.**
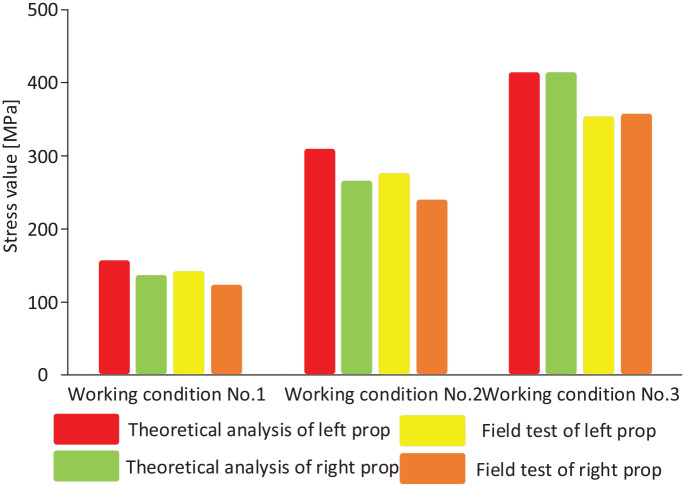
Comparison of stresses at the column sockets under the three working conditions.

In the simulation test bench, a steel frame is used to simulate the high-rigidity end bracket, a thin steel plate is used to simulate the roof of the coal mining face, and a weight is used to simulate roof load. The key research object is the hydraulic support. Therefore, a hydraulic support model is used to support the thin steel plate. The strain test data acquisition system measures the strain of the critical portion of the hydraulic support model under different working conditions. We compared field test data and results from theoretical analysis. Theoretical analysis results show that the stress differences are significant near the column socket of hydraulic support top beam. A comparison of the theoretical and field test results under the three working conditions is shown in Figure 16.

Under working condition no. 2, the stress at the column socket reaches 408.28 MPa. The stress at the left column socket is higher than that at the right due to offset loading. The stress at the upper left of the middle rib plate of the top beam is relatively high, reaching 308.2 MPa, while that near the column socket at the right side of the beam is 264.52 MPa, resulting in a difference of 43.68 MPa. Compared to working condition no. 1, working condition no. 2 results in an increase of 20.35 MPa in the stress difference between the right and the left column sockets, which is mostly due to increased load offset. Under working condition no. 3, the stress around the column socket reaches 412.97 MPa. Roof load, instead of offset load, acts mostly on the top beam. As a result, the stresses on the column sockets on the left side and right side are essentially identical. The stress on the upper part of the middle rib plate of the top beam is significant, at 311.5 MPa.

**Figure 17.**
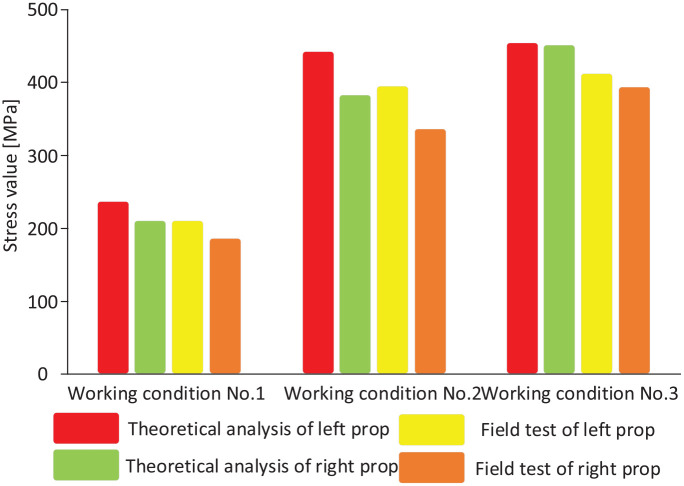
Stress comparison at the pinhole of the base under the three working conditions.

The theoretical analysis shows that stresses are concentrated on the pin connecting the column cylinder and base. We also found that large deformations should occur at the base. A comparison of the theoretical analysis results and field test results is shown in Figure 17.

Under working condition no. 2, the stress on the pinhole connecting the column and the base reaches 351.16 MPa with a deformation of 0.21922 mm. The deformation at the pinhole that connects the connecting rod and the base is 0.12284 mm. Under working condition no. 1, the stress on the pinhole connecting the column and the base increased by 163.44 MPa, and the deformation increased by 0.10756 mm, whereas the deformation at the pinhole connecting the connecting rod and the base increased by 0.05853 mm. Unlike in working condition nos. 1 and 2, working condition no. 3 resulted in similar stress-concentrated zones and deformations on both the left and right sides of the base. This is mostly because the top beam load imposed on the roof is not offset. Under working condition no. 3, the stress on the pinhole connecting the column and the base is 361.86 MPa with a deformation of 0.25499 mm. The deformation at the pinhole connecting the rear connecting rod and the base reaches 0.1508 mm.

**Figure 18.**
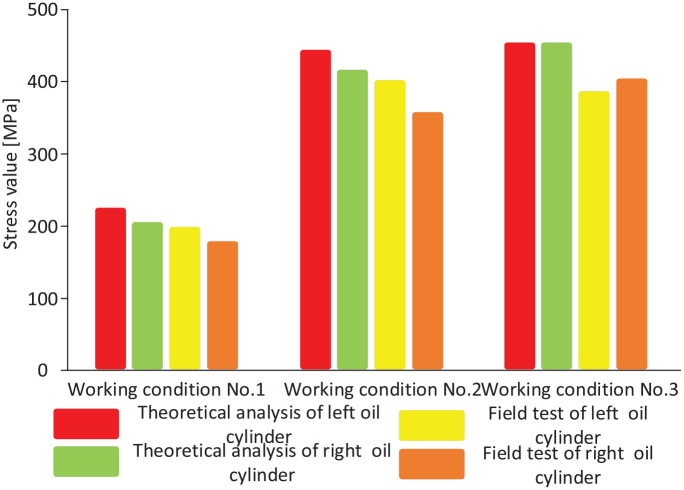
Comparison of stresses on the column cylinder block under the three working conditions.

A comparison of the column cylinder block stresses obtained from theoretical analysis and field tests under the three working conditions is shown in Figure 18.

Under working condition no. 2, the maximum equivalent stress on the column cylinder body reaches 443.76 MPa with a deformation of 2.0461 mm. Under the effects of offset load, the maximum equivalent stress difference and deformation discrepancy between the left and right column cylinders are 28.51 MPa and 0.2103 mm, respectively. Under working condition no. 3, the maximum equivalent stress on the column cylinder block reaches 453.78 MPa with a deformation of 2.4103 mm. Since the load under working condition no. 3 is not offset, the maximum equivalent stresses and the deformations on the left and right column cylinders are essentially the same.

**Figure 19.**
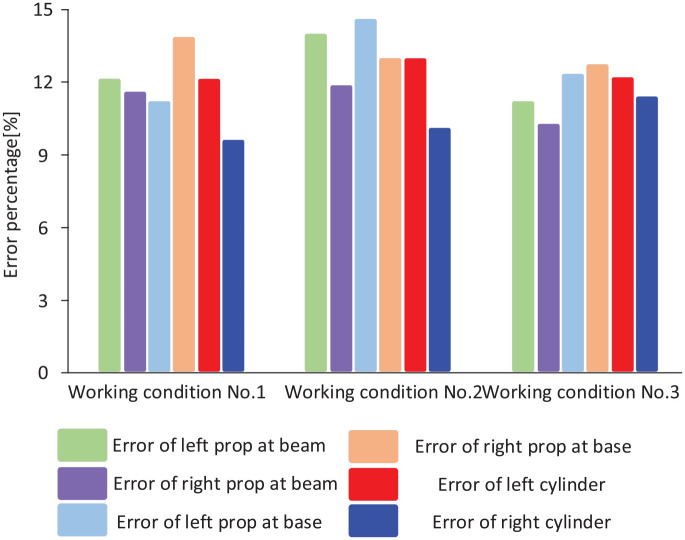
Comparison of errors from theoretical analysis and experiment for each key component under the three working conditions.

A comparison of errors from theoretical analysis and field tests can be found in Figure 19.

Under the three working conditions, theoretical analysis errors and field test errors for all key locations are less than 15%. This confirms the accuracy of our theoretical approach, which can be promoted for further practical applications.

## Conclusion

Simulations and field tests were conducted to investigate the mechanical characteristics and stiffness of key components of a hydraulic support group under different working conditions. The following conclusions are drawn from the study:

1. Loads and offset loads experienced by the hydraulic support vary with location along the working face. Supports near the middle of the working face had larger loads on their top beam, while the offset load experienced was reduced.
2. Compared to working condition no.1, working condition no. 2 yields a significant increase in stresses and deformations at key locations, indicating the deteriorating working conditions of the hydraulic support. Thus, a high-strength rib plate should be soldered at the pinholes connecting the column cylinder and the base, and the roof and the column cylinder, to meet the strength and stiffness requirements.
3. Under all the three working conditions, theoretical analysis errors and field test errors were less than 15% for all key locations. The result demonstrates the accuracy of our proposed method, which will be useful in hydraulic support structure design and analysis in the future.
